# Pistachio leaf waste transformed into a gut-targeted bioactive phytocomplex

**DOI:** 10.1016/j.isci.2025.113345

**Published:** 2025-08-14

**Authors:** Mariarita Spampinato, Salvatore Furnari, Laura Siracusa, Giuseppe A. Malfa, Alfio Distefano, Enrico La Spina, Massimo Gulisano, Pio Maria Furneri, Virginia Fuochi, Ignazio A. Barbagallo

**Affiliations:** 1 Department of Biomedical and Biotechnological Sciences, University of Catania, via Santa Sofia 97, 95124 Catania, Italy; 2 Department of Drug and Health Sciences, University of Catania, V.le A. Doria 6, 95125 Catania, Italy; 3 Institute of Biomolecular Chemistry, National Research Council (ICB-CNR), via Paolo Gaifami 18, 95126 Catania, Italy

**Keywords:** Health sciences, Nutrition, Microbiology, Phytochemistry

## Abstract

The human gut microbiota is integral to host physiology, influencing immune responses, metabolic regulation, and digestion. Disruptions in this microbial community are linked to inflammatory and metabolic disorders. In this study, we explored the potential of pistachio leaf extract (PLE), a polyphenol-rich natural product, to support gut health. The extract, obtained from *Pistacia vera* leaves harvested in Sicily, was chemically characterized and assessed for prebiotic-like, antimicrobial, antioxidant, and anti-inflammatory effects. It promoted the growth of beneficial gut bacteria, inhibited pathogenic strains, and exhibited marked antioxidant activity. In patient-derived intestinal organoids, the extract reduced inflammation and enhanced antioxidant defenses, with no detectable cytotoxicity in human colon cells. These results suggested that PLE is a safe and multifunctional compound that may contribute to intestinal homeostasis, offering promise for its inclusion in future functional food formulations aimed at promoting gastrointestinal health.

## Highlights

• Pistachio leaf extract (PLE) promotes probiotic growth and prolongs exponential phase
• PLE and probiotic supernatant enhance antimicrobial activity against pathogens
• PLE reduces inflammation and boosts antioxidant genes in intestinal organoids
• Sustainable reuse of pistachio leaf waste as gut-targeted functional ingredient

## Graphical abstract

**Figure:**
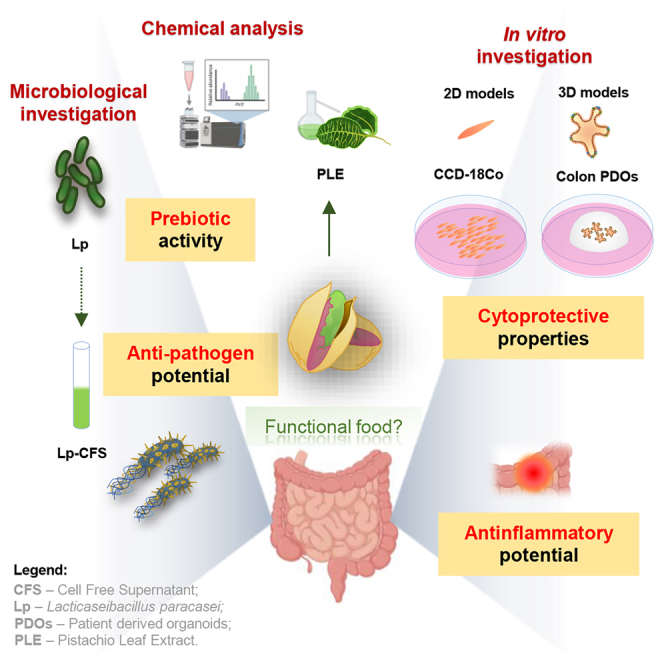

## Introduction

The increasing global focus on healthy lifestyles and balanced diets emphasizes the importance of preventing intestinal dysbiosis and related pathologies.^1^ Simultaneously, the growing world population and rising resource demands necessitate innovative strategies for optimizing agri-food waste management, contributing to more sustainable consumption practices. Among underutilized by-products of fruits and vegetables, materials such as leaves, seeds, and husks hold high potential for recovering bioactive functions, promoting both food and environmental sustainability.^2^^,^^3^ Food wastes play an important role also for dietary application, as certain agricultural by-products exhibit significant nutritional and cytoprotective properties, making them ideal candidates for functional food applications, complementing traditional therapeutic strategies.^3^^,^^4^

In this context, pistachio (*Pistacia vera* L.), globally appreciated for the nutritional value of its nuts, has become a major subject of research, particularly concerning the bioactivities of its edible nut and green hull. These plant parts have been extensively investigated for their lipid-lowering, antioxidant, and microbiota-modulating effects.^5^^,^^6^^,^^7^^,^^8^^,^^9^ Conversely, the leaves of *P. vera*, despite being a readily available agricultural by-product, remain underexploited in food and pharmaceutical applications. A growing body of literature has demonstrated that *Pistacia* leaves, including those from *P. vera, Pistacia atlantica, Pistacia lentiscus*, and *Pistacia terebinthus*, are rich in polyphenolic compounds (e.g., gallic acid, myricetin derivatives, quercetin glycosides) and exhibit potent antioxidant, antimicrobial, and anti-inflammatory properties.^10^^,^^11^^,^^12^ For instance, ethanolic extracts from *P. vera* leaves have been successfully used as bakery additives, increasing bread shelf life and improving its antioxidant profile.^10^ Moreover, extracts from *P. atlantica* and *P. lentiscus* have shown higher bioactivities than synthetic preservatives like ascorbic acid, particularly when obtained through optimized methods such as ultrasound-assisted or supercritical fluid extraction. Interestingly, *P. vera* leaf extracts have also been proven active against a wide spectrum of pathogens, including *Staphylococcus aureus, Enterococcus faecalis*, and *Klebsiella pneumoniae*, while maintaining low cytotoxicity on normal cells, underscoring their suitability for food applications.^11^ Unlike nuts and hulls, which are already part of the human diet, pistachio leaves represent a sustainable source of bioactive molecules that align with circular economy principles and support the valorization of agri-food waste. These compounds meet the evolving criteria of functional food ingredients, namely naturally occurring bioactives capable of providing beneficial physiological effects beyond basic nutrition, particularly in gut health, inflammation, and oxidative stress management.^13^^,^^14^ Based on these considerations, *Pistacia* leaf extracts can be considered promising candidates for incorporation into functional foods or dietary supplements aimed at supporting human health through preventive nutritional strategies, rather than through therapeutic or pharmacological intervention. Their long-standing use in traditional medicine further reinforces their potential for safe application in food systems.^15^^,^^16^

In parallel, scientific research over the past decades has underscored the role of gut microbiota in numerous physiological processes, including metabolic, digestive, and immune functions.^17^^,^^18^ Dysbiosis in the microbiota has been linked to autoimmune disorders, metabolic diseases, neurological conditions, and cancer, such as inflammatory bowel diseases and colorectal cancers.^19^^,^^20^ Given the critical role of the gut microbiota in human health, identifying natural bioactive molecules capable of enhancing its functions has become a strategic goal. Derivatives from *Pistacia* align well with this objective, demonstrating favorable effects on microbiota balance. Effectively, *Pistacia* species have been employed in traditional medicine since antiquity to treat a heterogeneous group of diseases,^16^ due to its numerous bioactive molecules, such as polyphenols, gallic acid derivatives, tannins, and numerous secondary metabolites.^21^^,^^22^ Indeed, studies in models of obesity and type 1 diabetes have shown pistachio’s ability to restore microbial populations, promoting beneficial bacteria such as *Bifidobacterium* and Lactobacilli.^6^^,^^7^ Advanced *in vitro* models, such as patient-derived organoids (PDOs), offer a promising solution to overcome the limitations of *in vivo* models by providing highly representative systems of the human intestinal niche.^23^

Considering these, this study aimed to evaluate the potential of pistachio leaf extract (PLE) as a functional food ingredient by systematically analyzing its biological activities relevant to intestinal health. Specifically, the research focused on (1) characterizing the compositional profile to identify its bioactive components; (2) assessing its prebiotic-like properties by examining the stimulation of beneficial gut bacteria such as *Lacticaseibacillus paracasei*; (3) determining its antimicrobial activity against clinically relevant pathogens commonly associated with human infections (including *S*. *aureus*, *Escherichia coli*, *Pseudomonas aeruginosa*, *K*. *pneumoniae*, *E*. *faecalis*, *Streptococcus agalactiae*, etc.), and evaluating possible synergistic effects with probiotic metabolites; (4) quantifying its antioxidant capacity to mitigate oxidative stress; and (5) investigating its anti-inflammatory properties in advanced 3D intestinal PDO models. A key innovative aspect of this functional food lies in the valorization of pistachio leaves, an agricultural by-product typically discarded, thus aligning with principles of sustainability and circular economy. By integrating both traditional microbiological techniques and cutting-edge organoid culture systems, this study seeks to establish a comprehensive understanding of PLE’s therapeutic potential and its practical applicability as a functional food for gut health management.

## Results

### Compositional analysis of PLE

The detailed composition of PLE extract was reported in Tables 1 and 2. The crude extract was characterized by the presence of a multitude of polyphenols; among the 17 peaks detected and tentatively identified, the presence of numerous flavonols stands out, such as galloyl and glycosyl derivatives of flavonols quercetin and myricetin. A member of the anacardic acid family (C17) was also detected in PLE extract.

**Table 1:** Specialized metabolites (peak number, retention time, tentative identification, and diagnostics) identified in the pistachio leaves object of this study

Peak	Rt, min	Tentative identification	Molecular formula	Exact mass, calculated^a^	Exact mass, experimental as (M-H)^b^
1	4.639	gallic acid^c^	C_7_H_6_O_5_	170.0215	169.08
2	17.966	quercetin di-hexoside	C_27_H_30_O_17_	626.1483	625.51
3	18.646	quercetin galloyl-hexoside 1	C_28_H_24_O_16_	616.1064	615.00
4	18.944	quercetin hexoside derivative	–	–	493.29
5	19.443	quercetin galloyl-hexoside 2	C_28_H_24_O_16_	616.1064	615.07
6	19.690	quercetin galloyl-hexoside 3	C_28_H_24_O_16_	616.1064	615.84
7	20.028	quercetin hexoside-deoxyhexoside	C_27_H_30_O_17_	610.1533	609.92
8	20.690	quercetin glucuronide	C_21_H_18_O_13_	478.0747	477.07
9	20.965	myricetin hexoside 1	C_21_H_20_O_13_	480.0903	479.00
10	21.193	myricetin hexoside 2	C_21_H_20_O_13_	480.0903	479.00
11	21.257	anacardic acid C17:1	C_24_H_38_O_3_	374.2820	373.50
12	21.755	quercetin 3-*O*-glucoside^c^	C_21_H_20_O_12_	464.0954	463.58
13	21.887	myricetin deoxyhexoside	C_21_H_20_O_12_	464.0954	463.17
14	22.846	myricetin galloyl glucuronide	C_28_H_22_O_18_	646.0806	645.09
15	23.067	myricetin galloyl hexoside	C_28_H_24_O_17_	632.1013	631.58
16	23.481	myricetin galloyl deoxyhexoside	C_28_H_24_O_16_	616.1064	615.25
17	24.510	myricetin digalloyl deoxyhexoside	C_35_H_28_O_20_	768.1174	767.17

Retention times refer to Uv-vis/DAD analyses reported in raw data files.

a Single isotope version, from https://www.sisweb.com/referenc/tools/exactmass.htm.

b See raw data files for individual mass spectra.

c Identified with the corresponding commercial analytical standard.

**Table 2:** Content of individual polyphenols, expressed in mg/100 mg extract, in the pistachio leaves object of this study

Peak	Rt, min^a^	Compound	mg/100 mg
1	4.639	gallic acid	2.681
2	17.966	quercetin di-hexoside	0.059
3	18.646	quercetin galloyl-hexoside 1	0.070
4	18.944	quercetin hexoside derivative	0.611
5	19.443	quercetin galloyl-hexoside 2	0.067
6	19.690	quercetin galloyl-hexoside 3	0.062
7	20.028	quercetin hexoside-deoxyhexoside	0.196
8	20.690	quercetin glucuronide	0.390
9	20.965	myricetin hexoside 1	0.038
10	21.193	myricetin hexoside 2	0.032
11	22.257	anacardic acid C17:1	0.144
12	21.755	quercetin 3-*O*-glucoside	0.098
13	21.887	myricetin deoxyhexoside (myricitrin)	0.135
14	22.846	myricetin galloyl glucuronide	0.090
15	23.067	myricetin galloyl hexoside	0.028
16	23.481	myricetin galloyl deoxyhexoside	0.012
17	24.510	myricetin digalloyl deoxyhexoside	0.011
–	–	total polyphenols	4.724
–	–	phenolic acids (gallic + anacardic)	2.825
–	–	quercetin derivatives	1.553
–	–	myricetin derivatives	0.346

a As mean of three replicates.

### Prebiotic-like activity of PLE

**Figure 1:**
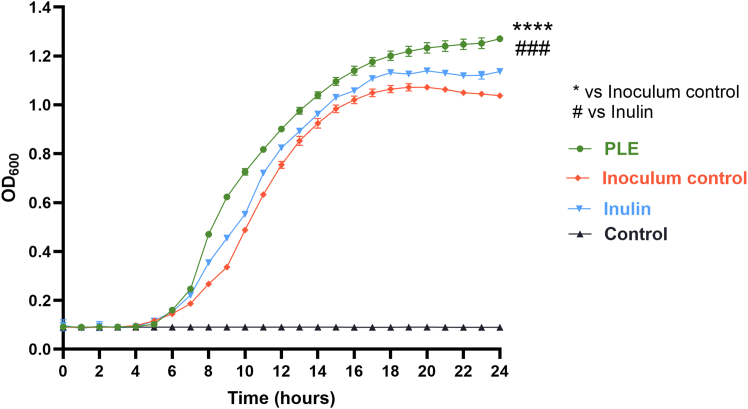
Prebiotic-like activity of PLE Each point represents spectrophotometric readings at OD_600_ taken every 30 min over a 24-h period. The green curve indicates bacterial growth in the presence of 200 μg/mL of PLE. The blue curve corresponds to growth in the presence of 0.02% (w/v) inulin, used as a recognized prebiotic control. The orange curve shows growth in MRS broth without additional compounds (inoculated control), while the black curve represents the negative control (non-inoculated MRS broth, to confirm medium sterility). *n* = 3 technical replicates from 3 biological replicates of the bacterial strain. The data were expressed as mean ± SD (∗∗∗∗^, ####^*p* < 0.0001).

As shown in Figure 1, PLE demonstrated a significantly enhanced effect on the growth of *L. paracasei* compared to both the inoculum control de Man, Rogosa, and Sharpe (MRS only) and inulin, a recognized prebiotic compound. This effect was particularly evident from the increased growth observed in the green curve (PLE treatment), which began to diverge approximately 6 h post-inoculation, coinciding with the onset of the bacterial logarithmic phase. In contrast, the orange curve (inoculum control) reached its growth plateau at approximately 16 h, and the blue curve (inulin) plateaued at around 18 h. Notably, the PLE-treated culture reached its plateau at 20 h, indicating a sustained stimulatory effect. These findings suggested that PLE not only enhanced bacterial proliferation but also prolonged the exponential phase relative to both controls. The superior performance of PLE compared to inulin highlighted its potential as an effective prebiotic-like candidate.

### Antibacterial activity of Lp-CFS and PLE on the main pathogens’ strains

To test the antimicrobial activity of *L. paracasei*-cell-free supernatant (Lp-CFS), PLE, and their combination, the substances were tested against six pathogens: *P. aeruginosa*, *S. aureus*, *E. coli*, *E. faecalis*, *K. pneumoniae*, and *S. agalactiae*. As shown in Table 3, antimicrobial activity was observed against all bacteria except for PLE against *E. coli*. Explicitly, when comparing the activity of Lp-CFS to PLE, both substances exhibited comparable activity against all pathogens, apart from a stronger activity of PLE against *S. aureus*. In contrast, Lp-CFS demonstrated superior activity against *E. coli* compared to PLE. Notably, the combination of Lp-CFS+PLE yielded remarkable results, demonstrating enhanced antimicrobial activity against all tested pathogens when compared to either substance alone. These results highlighted the antimicrobial properties of both PLE and Lp-CFS, with their effects being synergistically enhanced when combined.

**Table 3:** Antibacterial activity

Bacterial strain^a^	PLE	Lp-CFS	PLE+Lp-CFS
*P. aeruginosa* ATCC27853	18.0 ± 1.0	16.1 ± 0.2	20.0 ± 0.2
*S. aureus* ATCC29213	22.5 ± 0.5	12.2 ± 0.3	25.0 ± 0.1
*E. coli* ATCC25922	≤6	10.1 ± 0.2	12.0 ± 0.3
*E. faecalis* ATCC29212	30.5 ± 0.5	30.1 ± 0.1	35.0 ± 0.1
*K. pneumoniae* ATCC700603	11.0 ± 0.5	14.1 ± 0.2	16.0 ± 0.2
*S. agalactiae* ATCC2134	22.0 ± 0.5	21.3 ± 0.2	26.0 ± 0.3

a Results from agar well-diffusion test of PLE, Lp-CFS, and Lp-CFS+PLE against six pathogens. The results were obtained using a caliper and were expressed in mm ± SD. *n* = 3 technical replicates from 3 biological replicates for each strain.

### Viability of 2D culture

The viability of the CCD-18Co cell line following treatment with Lp-CFS was assessed to determine its prospective cytotoxicity. The 3-(4,5-dimethylthiazol-2-yl)-2,5-diphenyltetrazolium bromide (MTT) assay results demonstrated that Lp-CFS, across all tested concentration levels, did not induce any notable cytotoxic effects on the CCD-18Co cell line. The viability of the cells remained consistently above 90%, regardless of the concentration of Lp-CFS applied.

**Figure 2:**
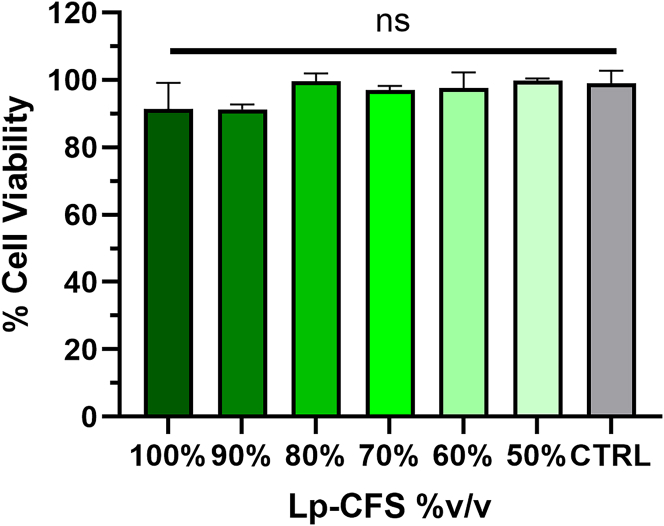
Evaluation of cytotoxicity of 2D culture (CCD-18Co) The cell viability was evaluated after 24-h exposure to decreasing concentrations of Lp-CFS, expressed as % v/v. “CTRL” referred to untreated cells incubated with standard growth medium only. Data were expressed as mean ± SD; *n* = 3 technical replicates from 4 biological replicates. Statistical analysis was performed using two-way ANOVA followed by Tukey’s multiple comparison test. No statistically significant differences were observed among the groups (^ns^*p* > 0.05).

As shown in Figure 2, a detailed analysis revealed no statistically significant differences in cell viability between the treated groups and the untreated control group. These findings strongly indicate that Lp-CFS is cytocompatible with human colon fibroblasts, even at high concentrations, supporting its potential as a safe and viable candidate for food applications targeting intestinal health.

This lack of cytotoxicity further highlights the guarantee of Lp-CFS in applications that require preserving the structural and functional integrity of intestinal cells.

### Evaluation of antioxidant efficacy of PLE: total phenolic content and total flavonoid content

The analysis of PLE by Folin-Ciocâlteu method revealed a remarkable amount of phenolic compounds equal to 223.80 ± 1.33 mg 3,4,5-trihydroxybenzoic acid (gallic acid) equivalent (GAE)/g extract (Table 4).

**Table 4:** Total phenolic (TPC) and flavonoid content (TFC) of PLE, expressed in mg GAE/g and mg CE/g extract, respectively

	Total polyphenols (mg GAE/g extract)	Total flavonoids (mg CE/g extract)
PLE	223.80 ± 1.33	74.30 ± 0.78

The content of total flavonoids in PLE determined by aluminum chloride assay, as reported in Table 4, highlighted a considerable presence of flavonoid derivatives equal to 74.30 ± 0.78 mg catechin equivalent (CE)/g extract.

### Evaluation of antioxidant efficacy of PLE: 2,2-Diphenyl-1-picrylhydrazyl assay

**Figure 3:**
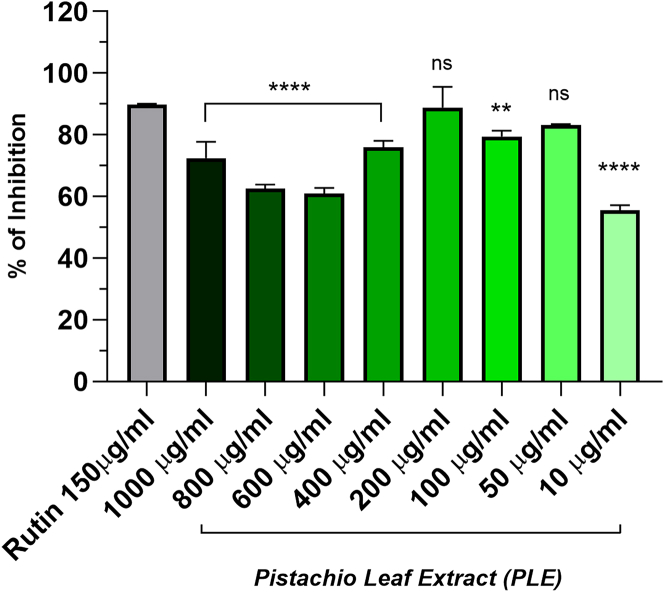
Dose-dependent DPPH radical scavenging activity of PLE DPPH assay was performed to evaluate the anti-scavenger properties of scalar concentrations of PLE. Rutin 150 μg/mL was considered as positive standard. Statistical analysis was performed using one-way ANOVA test with Tukey’s multiple comparison test. The data were expressed as mean ± SD; *n* = 3 technical replicates from 4 biological replicates (^ns^*p* > 0.05, ∗*p* < 0.05, ∗∗*p* < 0.01, ∗∗∗∗*p* < 0.0001).

As shown in Figure 3, the effect of the extract at different concentrations—1000, 800, 600, 400, 200, 100, 50, 10 μg/mL—was investigated. All concentrations demonstrated an inhibition of DPPH radicals higher than 50%, with a particular mention for 200 μg/mL, that resulted comparable to the standard control Rutin 150 μg/mL.

### Cyto-compatibility of PLE toward *in vitro* intestinal models

**Figure 4:**
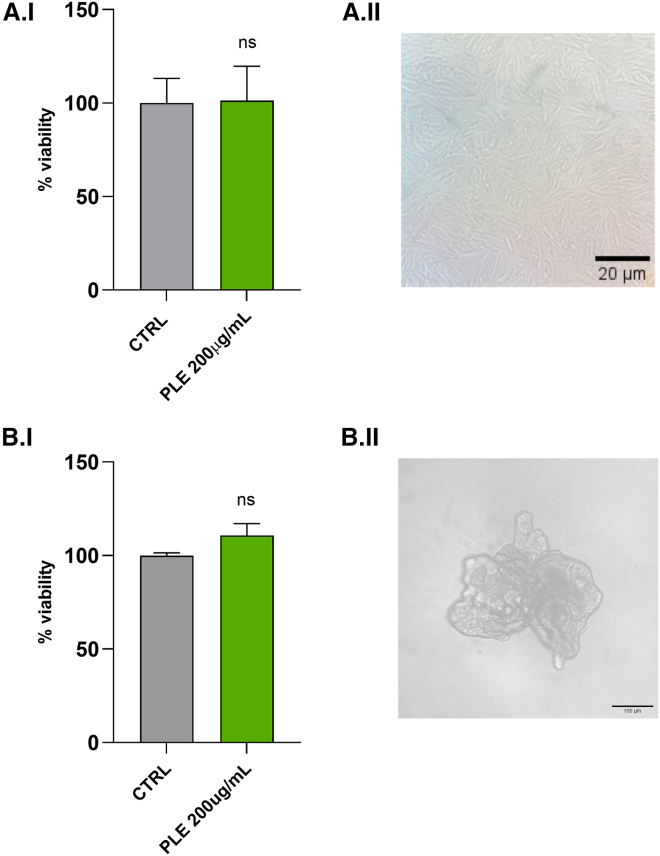
Effect of PLE on the viability of normal human colon fibroblasts (CCD18Co) and patient-derived organoids (PDOs) (A.I) Cell viability in CCD18Co cells treated with 200 μg/mL PLE for 24 h, compared to untreated controls (CTRL); (A.II) representative bright-field micrograph of CCD18Co cells following 24-h treatment with 200 μg/mL PLE, showing normal fibroblast morphology without detectable cytotoxic effects (20x magnification, scale bars, 20 μm). *n* = 3 technical replicates from 4 biological replicates (^ns^*p* > 0.05); (B.I) viability in PDOs exposed to 200 μg/mL PLE for 24 h. Like CCD18Co cells, no statistically significant change (ns) was observed compared to untreated PDOs; (B.II) representative bright-field micrograph of PDOs after treatment, demonstrating intact organoid structure and morphology, indicative of preserved viability (10x magnification, scale bars, 100 μm). *n=*3 technical replicates from 3 biological replicates (^ns^*p* > 0.05). All experiments were performed under the same conditions of culture and incubation. The data were expressed as mean ± SD (^ns^*p* > 0.005). Statistical analysis was performed by Student’s t test with two-tailed *p*-value calculation.

Since the aim of the study was to evaluate the role of PLE in the intestinal niche, it was necessary to exclude any possible cytotoxic effect. In this regard, both 2D and 3D models were treated with PLE for 24 h (200 μg/mL). Viability data obtained on CCD18Co cell line and colon PDOs confirmed that PLE did not show any cytotoxic effect after 24 h of treatment. In addition, PLE seems to exert a slight effect of proliferative enhancement on PDOs, as shown in Figure 4, compared to the untreated control.

### Anti-inflammatory features of PLE

**Figure 5:**
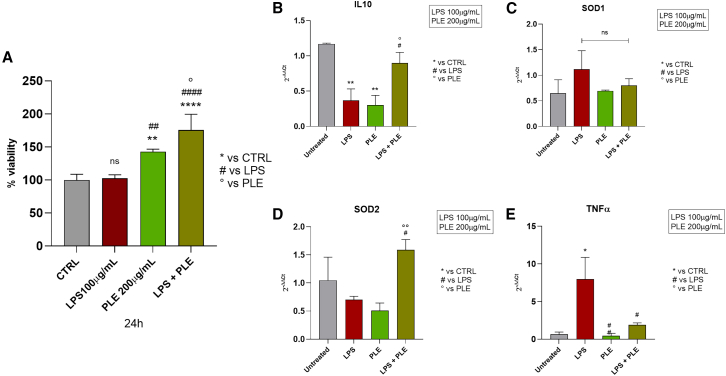
Evaluation of anti-inflammatory effect of PLE on LPS-induced inflammation (A) MTT viability assay on PDOs after 24-h treatment of PLE after LPS-induced inflammation; *n* = 3 technical replicates from 2 biological replicates; mRNA expression of (B) IL-10, (C) SOD1, (D) SOD2, and (E) TNFα. PDOs were treated with LPS 100 μg/mL for 1 h and with PLE 200 μg/mL for 24 h. The relative genes expressions were quantified using the 2^−ΔΔCt^ method. Statistical analysis was performed using one-way ANOVA test with Tukey’s multiple comparison test. The data were expressed as mean ± SD; *n* = 3 technical replicates from 1 biological replicate (^ns^*p* > 0.05, ∗, ^○^, #*p* < 0.05, ∗∗, ^○○^, ##*p* < 0.01, ∗∗∗∗, ^####^*p* < 0.0001).

The results presented in the histograms in Figure 5 demonstrate the effects of PLE on viability and inflammatory gene expression in a PDOs model exposed to lipopolysaccharide (LPS) 100 μg/mL. The viability assay revealed that while treatment with LPS did not significantly affect cell viability, PLE (200 μg/mL) restored it, exceeding the levels observed in the other groups. Analysis of gene expression revealed that LPS significantly downregulated anti-inflammatory interleukin-10 (IL-10) expression and upregulated pro-inflammatory tumor necrosis factor alpha (TNF-α) levels, highlighting the activation of the inflammatory response. Treatment with PLE alone maintained basal expression levels of IL-10 and TNF-α, while the combination of LPS and PLE significantly increased IL-10 expression and reduced TNF-α levels compared to LPS. Additionally, the expression of oxidative stress markers superoxide dismutase 1 (SOD1) and SOD2 remained unchanged under LPS treatment, whereas co-treatment with PLE markedly upregulated SOD2, suggesting enhanced antioxidant activity. These findings collectively indicate that PLE mitigates LPS-induced inflammation and oxidative stress.

### Evaluation of PLE efficacy toward an elementary model of *in vitro* intestinal niche

**Figure 6:**
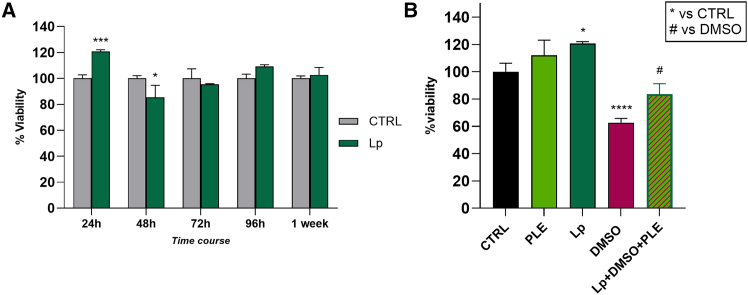
Evaluation of PLE cytoprotective efficacy toward an elementary model of *in vitro* intestinal niche (A) MTT assay showing percentage of viability in time course of PDOs co-cultured with *L. paracasei* at different time points (24 h, 48 h, 72 h, 96 h, and 1 week); *n* = 3 technical replicates from 3 biological replicates. (B) MTT assay of PDOs treated with PLE 200 μg/mL, Lp (1.5 × 10^7^ CFU/mL), DMSO 5% v/v for 24 h. Statistical analysis was performed using one-way ANOVA test with Tukey’s multiple comparison test. The data were expressed as mean ± SD; *n* = 3 technical replicates from 3 biological replicates (∗^,^^*#*^*p* < 0.05, ∗∗∗*p* < 0.001, ∗∗∗∗*p* < 0.0001).

As shown in Figure 6A, a preliminary evaluation of *L. paracasei* (Lp) (1.5 × 10^7^ CFU/mL) effects toward PDO culture was performed. Particularly, the data obtained demonstrated that Lp did not affect PDO viability till 1 week of co-culture. Afterward, the potential efficacy of Lp and PLE against a cytotoxic injury was evaluated (Figure 6B). The results confirmed that both Lp and PLE treatments were non-toxic to PDOs, with Lp significantly enhancing cell viability compared to the untreated control. In contrast, the standard cytotoxicity model using dimethyl sulfoxide (DMSO) 5%v/v caused a marked reduction in viability, with a decrease of approximately 40% after 24 h. Remarkably, the combination treatment with Lp and PLE significantly reversed the cytotoxic effects promoted by DMSO, highlighting the cytoprotective properties of PLE and Lp against a cytotoxic insult, preserving intestinal cell integrity under stress conditions.

## Discussion

The valorization of *Pistacia vera* leaf waste into a bioactive functional ingredient aligns with the principles of the circular economy and sustainable agri-food practices.^2^^,^^3^^,^^24^^,^^25^^,^^26^^,^^27^ PLE, obtained from *P*. *vera* leaves, exemplifies this principle by converting agricultural waste into functional food ingredients with considerable biological potential.^5^^,^^9^^,^^28^^,^^29^^,^^30^ This study provided innovative evidence regarding the biological properties of PLE, emphasizing its prebiotic-like, antibacterial, antioxidant, and anti-inflammatory activities.

The enhanced growth of Lp in the presence of PLE, significantly outperforming the standard prebiotic inulin, confirmed its robust prebiotic-like activity. Although the term prebiotic-like” is not yet formally defined, it is often used to describe substrates that promote beneficial microbial growth and activity without meeting the full criteria for established prebiotics, such as selective fermentation.^31^ This strain was selected due to its documented probiotic efficacy and widespread inclusion in commercially available gut-health supplements.^32^^,^^33^^,^^34^^,^^35^ Furthermore, the unique dominance of Lactobacilli in specific body niches, such as the human vaginal microbiota, has been proposed as an evolutionary trait to maintain acidic pH and inhibit pathogen colonization, highlighting the broader ecological and health relevance of this genus beyond the intestinal niche.^36^

Although direct comparisons in the literature remain limited due to the scarcity of studies on *P. vera* leaves, similar effects have been observed with pistachio nuts and hulls, which modulate the gut microbiota by increasing *Lactobacilli* and *Bifidobacterium* populations.^7^ The inclusion of inulin as a positive control in our assays has now addressed a key methodological limitation, enabling clearer comparison and reinforcing the validity of our findings. Moreover, a recent human intervention trial demonstrated that pistachio consumption increased butyrate-producing taxa and promoted microbiota shifts associated with improved metabolic parameters, further supporting the gut-modulating potential of pistachio-derived ingredients.^37^^,^^38^ These findings reinforced the relevance of PLE as a potential prebiotic-like agent.

Furthermore, a panel of clinically relevant pathogens, including *P. aeruginosa*, *S. aureus*, *E. coli*, *K. pneumoniae*, *E. faecalis*, and *S. agalactiae*, was selected to investigate the antimicrobial potential of probiotic-derived CFSs in the context of dysbiosis across multiple anatomical sites. This broader approach was adopted to move beyond the extensively studied gut microbiota. The antimicrobial efficacy of CFSs and postbiotics against some of these pathogens has already been demonstrated in previous studies, including those by Fuochi *et al.*,^39^ which reported significant inhibitory effects against *S. aureus*, *E. coli*, and *K. pneumoniae*. These findings supported the rationale behind the selected panel and experimental design. Here, PLE showed antibacterial effects against pathogens such as *P. aeruginosa* and *E. faecalis*. Additionally, when combined with Lp-CFS, its antibacterial efficacy was greatly enhanced, indicating synergistic interactions. These results suggested that PLE exerts selective antimicrobial activity and may indirectly support microbial homeostasis, although its direct impact on complex microbiota composition remains to be elucidated. These pathogens are frequently implicated in opportunistic infections, including those associated with dysbiosis, antibiotic resistance, and compromised gut barrier function.^40^^,^^41^^,^^42^^,^^43^ Therefore, the observed antimicrobial effects of PLE suggested its role not only in pathogen suppression but also in the preservation of gut microbial balance, particularly under stress or inflammatory conditions.

PLE’s bioactivity was found to correlate with its rich polyphenolic profile, as confirmed by high-performance liquid chromatography with diode array detection (HPLC-DAD) and high-performance liquid chromatography with mass spectrometry (HPLC-MS) analyses. Compounds such as gallic acid and flavonoids are known for their antioxidant, antibacterial, and anti-inflammatory effects.^44^^,^^45^^,^^46^^,^^47^ These phenolics were suggested to exert protective effects through reactive oxygen species (ROS) scavenging, inhibition of bacterial enzymes, and suppression of pro-inflammatory cytokines. Quercetin derivatives, for instance, have been shown to inhibit LPS-induced nitric oxide production and suppress nuclear factor κB (NF-κB)-light-chain-enhancer of activated B cell activation in macrophages,^48^ while gallic acid disrupts bacterial membrane integrity and inhibits virulence factor expression.^49^ These mechanistic data provided a molecular rationale for the observed bioactivities of PLE.

Notably, this study identified the presence of anacardic acid in *P*. *vera* leaves, albeit in small quantities. Anacardic acid is a bioactive phenolic lipid predominantly found in cashew nut shells, known to modulate key biological processes such as intracellular signaling, inflammatory responses, and antimicrobial mechanisms. Its amphiphilic structure, characterized by a salicylic-acid-derived polar head and a hydrophobic alkyl tail, enables interactions with both lipid membranes and protein targets, contributing to its biochemical versatility. First report of the presence of alkylphenols in *Pistacia vera* dates back to 2009 with the work of Saitta et al. on pistachio kernels,^50^ followed by other studies on more conventional by-products from this species (hulls^51^ and shells^52^). The discovery of anacardic acid in pistachio leaves is particularly significant, as it highlighted the untapped potential of this matrix as a renewable and sustainable source of high-value bioactive molecules at the same time suggesting additional functional properties, such as antioxidant and antimicrobial activities, that could be exploited for food and pharmaceutical applications. This identification not only enriches the chemical profile of *Pistacia vera* leaves but also paves the way for exploring their broader utilization.

Additionally, a previous animal study demonstrated that pistachio extracts modulate lipid metabolism and improve plasma fatty acid composition by increasing monounsaturated fatty acids such as oleic acid,^53^ highlighting further systemic benefits of pistachio-derived ingredients.

PLE exhibited a clear capacity to mitigate oxidative stress, a significant factor for maintaining cellular homeostasis. In a three-dimensional (3D) inflammation model, the impact of PLE on the expression of *TNF-α*, *IL-10*, and both *SOD* isoforms was assessed. In these models using intestinal organoids, treatment with PLE resulted in a considerable reduction in pro-inflammatory markers such as TNF-α, while concurrently enhancing the expression of the anti-inflammatory cytokine IL-10. The increased expression of oxidative-stress-related genes, particularly *SOD2*, underscored its protective role against inflammation-induced oxidative damage. Notably, PLE selectively enhanced *SOD2* (mitochondrial superoxide dismutase) expression without affecting the cytosolic isoform *SOD1*, suggesting a mitochondrial-specific antioxidant action.

These findings were consistent with and expanded upon previous evidence supporting the anti-inflammatory activity of pistachio-derived extracts. Several studies had demonstrated that bioactive compounds from *Pistacia vera* L. influenced key inflammatory pathways, particularly through the modulation of NF-κB, a redox-sensitive transcription factor involved in the regulation of pro-inflammatory genes.^54^^,^^55^^,^^56^ Specifically, hydrophilic pistachio extracts were shown to downregulate NF-κB activity and suppress the expression of inducible nitric oxide synthase (iNOS), cyclooxygenase-2 (COX-2), and TNF-α.^57^^,^^58^^,^^59^ These effects might have been attributed to the ability of pistachio polyphenols to modulate redox-sensitive signaling pathways and attenuate nitrosative stress, thereby limiting nitric oxide (NO) production. Although further investigations were necessary to elucidate the precise mechanisms involved, these results highlighted the relevance of PLE in modulating both antioxidant and anti-inflammatory responses at the cellular level.

One of the study’s major strengths is the use of 3D PDOs, which replicate the cellular architecture and functionality of the human intestinal epithelium more accurately than traditional 2D models.^60^^,^^61^ PDOs provide a forceful platform to study the interactions between PLE and intestinal cells in a controlled yet physiologically relevant environment. The adoption of these models increases the translational ability of the findings while addressing ethical concerns associated with animal testing.

PLE’s potential applications as a functional food are strong, given its ability to modulate gut microbiota, reduce inflammation, and combat oxidative stress. Products derived from other *Pistacia* spp. have already been integrated into functional foods and supplements for their bioactive properties.^62^^,^^63^ Incorporating PLE into dietary formulations alongside probiotics like *L. paracasei* could create synergistic effects, providing a comprehensive approach to gut health management. Moreover, the sustainability of PLE production must be documented to support claims made in the introduction. Future efforts should include green extraction protocols, yield optimization, and solvent recovery strategies to minimize environmental impact. The scale-up of PLE extraction also presents challenges, particularly in maintaining batch-to-batch consistency and ensuring compliance with food-grade quality standards.

### Limitations of the study

Nonetheless, certain limitations must be acknowledged. The *in vitro* models used cannot fully replicate the complexity of human metabolism, including the biotransformation and bioavailability of PLE. Pistachio polyphenols are known to undergo extensive metabolism during digestion, resulting in a variety of low-molecular-weight catabolites that may exert biological effects distinct from their parent compounds.^64^ However, although pistachios have been safely consumed for millennia as part of the human diet, the use of leaf-derived polyphenol-rich extracts in functional foods represents an innovative application. To date, no specific studies have addressed the bioavailability, metabolism, or safety profile of PLE *in vivo*. Therefore, further research is warranted to define its pharmacokinetic behavior, effective dosing, and potential adverse effects under chronic or high-intake conditions.

### Conclusions

In summary, PLE emerged as a multifunctional ingredient with prebiotic-like, antibacterial, antioxidant, and anti-inflammatory properties. Through a combination of advanced *in vitro* models and detailed compositional analyses, we demonstrated PLE’s efficacy in modulating gut microbiota, supporting intestinal cell viability, and counteracting inflammatory and oxidative stress stimuli. The synergistic interactions observed with probiotic metabolites further suggested its potential for use in combined food formulations.

This study provided a proof of concept for the sustainable valorization of pistachio leaf waste in functional food applications. Future directions should include *in vivo* pharmacokinetic studies, safety and efficacy trials, and regulatory assessments. Addressing scale-up, standardization, and circular economy modeling will be essential to enable the real-world translation of this promising biowaste-derived ingredient.

## Resource availability

### Lead contact

Requests for further information and resources should be directed to and will be fulfilled by the lead contact, Virginia Fuochi (virginia.fuochi@unict.it).

### Materials availability

There are restrictions to the availability of PLE because of the lack of an external centralized repository for its distribution and our need to maintain the stock.

### Data and code availability

• Raw data have been deposited at Mendeley at https://doi.org/10.17632/3fcpn5zp8r.1 and are publicly available as of the date of publication.
• Data not deposited at https://doi.org/10.17632/3fcpn5zp8r.1 will be shared by the lead contact upon request.
• This paper does not report original code.
• Any additional information required to reanalyze the data reported in this paper is available from the lead contact upon request.

## Acknowledgments

Professor Virginia Fuochi was supported by the European Union – NextGenerationEU through the Italian Ministry of University and Research under PNRR – M4C2-I1.3 Project PE0000019 “HEAL ITALIA” (CUP E63C22002080006, University of Catania). The views and opinions expressed are those of the authors only and do not necessarily reflect those of the European Union or the European Commission. Neither the European Union nor the European Commission can be held responsible for them. The authors are grateful to Dr. Francesco Mugheddu (ICB-CNR) for his skillful assistance in providing MS raw data.

This research was funded by PIA.CE.RI. 2024–2026 Linea 1 FUMARACREB Prof. I.A.B.

## Author contributions

Conceptualization, I.A.B.; methodology, M.S., S.F., L.S., G.A.M., A.D., E.L.S., and V.F.; investigation, M.S., S.F., L.S., G.A.M., A.D., E.L.S., and V.F.; formal analysis, M.S., S.F., L.S., and G.A.M.; visualization, M.S., S.F., and V.F.; writing—original draft, M.S., S.F., L.S., G.A.M., P.M.F., and V.F.; writing—review & editing, M.S., S.F., G.A.M., V.F., and I.A.B.; supervision, P.M.F. and V.F.; validation, P.M.F. and V.F.; resources, M.G. and I.A.B.; funding acquisition, I.A.B.

## Declaration of interests

The authors declare no competing interests.

## Declaration of generative AI and AI-assisted technologies in the writing process

During the preparation of this work, the authors used ChatGPT (Open AI 4o) to improve readability and language. After using this tool, the authors reviewed and edited the content as needed and take full responsibility for the content of the published article.

## STAR★Methods

### Key resources table

**Table:** 

REAGENT or RESOURCE	SOURCE	IDENTIFIER
**Bacterial strains**		

*Lacticaseibacillus paracasei* subsp*. paracasei*	Commercial product	CNCM I-1572-LCDG
*Staphylococcus aureus*	ATCC	29213
*Escherichia coli*	ATCC	25922
*Pseudomonas aeruginosa*	ATCC	27853
*Streptococcus agalactiae*	ATCC	13813
*Klebsiella pneumoniae*	ATCC	700603
*Enterococcus faecalis*	ATCC	29212

**Biological samples**		

*Pistacia vera* L. leaves	This paper	No. 08/22

**Chemicals, peptides, and recombinant proteins**		

100 U/mL penicillin-100 U/mL streptomycin	Gibco	15070063
3-(4,5-dimethylthiazol-2-yl)-2,5-diphenyltetrazolium bromide (MTT)	Thermo Fisher Scientific Inc	L11939.06
Dimethyl sulfoxide (DMSO)	PanBiotech	P60-36720100
2,2-Diphenyl-1-picrylhydrazyl (DPPH)	Merck KGaA	D9132
Dulbecco’s Modified Eagle’s Medium/Nutrient Ham’s Mixture F-12 (DMEM/F-12) with 15 mM HEPES buffer	STEMCELL Technologies	# 36254
Dulbecco’s Phosphate Buffer Saline (DPBS)	Euroclone	ECB4004L
Eagle’s minimum essential medium (EMEM)	ATCC	30–2003
Fetal bovine serum	Gibco	10082147
Gentle Cell Dissociation Reagent	STEMCELL Technologies	# 100-0485
Inulin	ACEF	005521
Lipopolysaccharide from *E. coli*	Sigma-Aldrich	L2880
Man, Rogosa, and Sharpe (MRS) agar	Thermo Fisher Scientific Inc	CM0361
Man, Rogosa, and Sharpe (MRS) broth	Thermo Fisher Scientific Inc	CM0359
Matrigel gf red 10 mL phenol red free	Corning	356231
Muller-Hinton (MH) agar	Thermo Fisher Scientific Inc	CM0337
Muller-Hinton (MH) broth	Thermo Fisher Scientific Inc	CM0405
PowerUp™ SYBR™ Green Master Mix for qPCR	Applied Biosystems	A25742
Rutin	Merck KGaA	PHL89270
Sodium Dodecyl Sulfate (SDS)	F. Hoffmann-La Roche Ltd	11667289001
TRIzol™ Reagent	Invitrogen	15596018
trypsin–EDTA solution	Gibco	25300054
L-Glutamine (200 mM)	BioConcept Ltd.	5-10K00-H
Gallic Acid (3,4,5-trihydroxybenzoic acid)	Sigma-Aldrich	≥99%, 27645
Quercetin 3 - *O* - glucoside (3,3′,4′,5,7-Pentahydroxyflavone 3-β-glucoside)	Sigma-Aldrich	16654 (Supelco)
Myricetin 3-*O*-rhamnoside (3,3′,4′,5,5′,7-Hexahydroxyflavone 3-*O*-α-L-rhamnopyranoside)	Sigma-Aldrich	≥99%,91255
Ginkolic acid 17:1 (6-[(10Z)-Heptadecenyl]salicylic acid)	Sigma-Aldrich	55822

**Critical commercial assays**		

IntestiCult™ Organoid Growth Medium (Human)	STEMCELL Technologies	# 06010
Applied Biosystems™ High-Capacity cDNA Reverse Transcription Kit	Applied Biosystems	10400745
MycoStrip® kit	InvivoGen	rep-mys-50

**Deposited data**		

Mendeley Data	https://data.mendeley.com	https://doi.org/10.17632/3fcpn5zp8r.1

**Experimental models: Cell lines**		

Non-transformed human colon fibroblasts CCD-18Co	ATCC	CRL-1459
Intestinal patient derived organoids (PDOs)	Sato et al.^61^	https://doi.org/10.1038/nature07935

**Oligonucleotides**		

IL10; Fw: CCACAAGACAGACTTGCAAAAG Rv: AACAAGTTGTCCAGCTGATCC	This paper	Accession code NM_000572.3
SOD1; Fw: AAAGATGGTGTGGCCGATGT Rv: CAAGCCAAACGACTTCCAGC	This paper	Accession code NM_000454.5
SOD2; Fw: CTGGACAAACCTCAGC CCTAAC Rv: AACCTGAGCCTTGGA CACCAAC	This paper	Accession code NM_000636.4
TNFα; Fw: GCAACAAGACCACCACTTCG Rv: GATCAAAGCTGTAGGCCCCA	This paper	Accession code NM_000594.4
βactin; Fw: GCCTCGCCTTTGCCGAT Rv: AGGTAGTCAGTCAGGTCCCG	This paper	Accession code NM_001101.5

**Software and algorithms**		

ImageJ2(Fiji)	Open source	github.com/fiji/fiji
Prism	GraphPad, Dotmatics	www.graphpad.com
Chromeleon Chromatography Information Management System v.6.80	Thermo Fisher Scientific Inc	www.thermofisher.com
Thermo Scientific Xcalibur version 4.5	Thermo Fisher Scientific Inc	www.thermofisher.com

### Experimental model and study participant details

#### Bacterial strains

The strain L. *paracasei* subsp. *paracasei* (CNCM I-1572-LCDG), previously isolated from commercial probiotic product, was used. Bacterium was grown in MRS broth (Oxoid, Thermo Fisher Scientific Inc., Rodano (MI), Italy, CM0359) at 37°C for 48 h under microaerobic conditions using GasPak (BD GasPakTM 100 Systems) before proceeding with subsequent investigations.

Moreover, ATCC strains of pathogenic bacteria were used to assess antibacterial activity: *P. aeruginosa* ATCC 27853, *S. aureus* ATCC 29213, *E. coli* ATCC 25922, *E. faecalis* ATCC 29212, *K. pneumoniae* ATCC 700603, *S. agalactiae* ATCC 13813. Bacterial strains were grown in Muller-Hinton (MH) broth (Oxoid, Thermo Fisher Scientific Inc., Rodano (MI), Italy, CM0405) at 37°C overnight before proceeding with subsequent investigations.

All strains are available to Laboratory of Applied Microbiology, Department of Biomedical and Biotechnological Sciences, Università di Catania.

For this study, each participant provided informed consent by signing a statement declaring voluntary participation. The study procedures, conditions, and ethical guidelines strictly adhered to the World Medical Association’s Declaration of Helsinki (2013). Additionally, all participant data were treated anonymously and processed in accordance with the relevant Italian laws. Samples’ collection and experiments were approved by Ethics Committee 2 – Catania (I) under reference number prot. 601/C.E.).

#### CCD18Co cell line and culture maintenance

Non-transformed human colon fibroblasts CCD-18Co (CRL-1459), derived from 2.5 months old black female infant, were purchased from ATCC Company (Manassas, Virginia, United States). The cell line was authenticated by ATCC using STR (short tandem repeat) profiling. Cells were suspended in Eagle’s minimum essential medium (EMEM) (30–2003, ATCC Company). The culture medium contained 10% fetal bovine serum (FBS, Gibco, Cat. No. 10082147), 100 U/mL penicillin, and 100 U/mL streptomycin (Gibco, Cat. No. 15070063). At 80% confluency, cells were passaged (seeding passage 20) using a trypsin–EDTA solution (0.05% trypsin and 0.02% EDTA, Gibco, Cat. No. 25300054).

#### Intestinal patient-derived organoids (PDOs) isolation and culture maintenance

A biopsy of healthy mucosa from the transverse colon was obtained from a 48-year-old female patient. in the “Fondazione mediterranea G.B. Morgagni, Catania, Italy”. Biopsies’ processing and crypts isolation were performed following the methodological guidelines of Sato et al.^65^^,^^66^ Organoids were derived from biopsy of a female patient (aged 48) of transverse colon.

To let organoids properly develop *in vitro*, the mechanical and nutritional support was enabled by embedding crypts in matrigel (Matrigel Growth Factor Reduced Basement Membrane Matrix, Corning) domes. Once organoids cultures, from (PDOs) were obtained, they were subjected to passage every 7 days and medium was replaced every 48 h (maximum number of passages approximately equal to 9–10). Culture maintenance was performed using the IntestiCult Organoid Growth Medium kit (StemCell technologies) supplemented with penicillin/streptomycin.

### Method details

#### Plant collection, identification and extraction procedure

*P*. *vera* L. leaves were harvested in Bronte (Sicily, Italy) in September 2022 and authenticated by the pharmaceutical botanist G.A. Malfa. Plant material was collected with landowner permission (I.A.B.) and did not require specific institutional approval. A voucher specimen (No. 08/22) was deposited in the herbarium of the Department of Drug and Health Sciences. Fresh leaves were dried at 40 °C for 72 h in a ventilated oven. 25 g of ground plant material was extracted at 80 °C in distilled water for 1 h (ratio 1:20). The extraction process was repeated three times; then, the pooled solution was filtered with Whatman filter paper N°4 and evaporated to dryness with a rotatory evaporator, obtaining about 4.42 g of dry extract (17.7%). The extraction conditions were selected by integrating empirical knowledge from traditional practices, peer-reviewed literature, and the physicochemical properties of key bioactive constituents of *Pistacia vera* (e.g., polarity and thermal stability), with a focus on sustainability and cross-disciplinary integration.^10^^,^^67^

#### Determination of total phenolic (TPC) and total flavonoid content (TFC)

The amount of total phenolic and flavonoid compounds in the extract was determined spectrophotometrically using the Folin–Ciocâlteu method and aluminum chloride assay, by comparison with a calibration curve of a known quantity of gallic acid (GA) and catechin (C) respectively.^68^ Results were expressed as mg of GA equivalent/g of extract for TPC and mg of C equivalent/g extract. Data were obtained from three independent determinations.

#### HPLC/Uv-vis-DAD and HPLC/ESI-MS specialized metabolic profile of PLE

Chromatographic analyses were carried out on an Ultimate3000 UHPLC focussed instrument equipped with a binary high-pressure pump, a Photodiode Array detector, a Thermostatted Column Compartment and an Automated Sample Injector (Thermo Fisher Scientific, Inc., Milan, Italy). Collected data were processed through a Chromeleon Chromatography Information Management System v. 6.80. Chromatographic runs were performed using a reverse-phase column (Gemini C18, 250 x 4.6 mm, 5 μm particle size, Phenomenex Italia s.r.l., Bologna, Italy) equipped with a guard column (Gemini C_18_ 4 x 3.0 mm, 5 μm particle size, Phenomenex Italia s.r.l., Bologna, Italy).

Components of the PLE were eluted using the same solvent system, elution gradient, flow rate, injection volume and quantitation method as detailed by Bouabidi et al*.*^69^ To unambiguously identify the chromatographic signals and/or to confirm peak assignments, UPLC/ESI/MS analyses were also performed on the extract employing a Vanquish UPLC System equipped with a quaternary high-pressure pump F (VF-P20-A), a photodiode array detector (VC-D11-A), a thermostated column compartment and an automated sample injector (VF-A10-A) (Thermo Fisher Scientific, Inc., Milan, Italy) coupled to a TSQ Fortis Plus Mass spectrometer. Collected data were processed through the software Thermo Scientific Xcalibur version 4.5. The chromatographic method discussed above was adapted to a reverse-phase 10 cm column (Luna Omega C18, 100 × 2.1 mm, 1,6 μm particle size, Phenomenex Italia s.r.l., Bologna, Italy); while the MS method has been set with a scan-range (m/z) of 150–1500 in negative (2200 V) mode, Q1 resolution of 0.7 (FWHM) and 20 eV as source fragmentation; the ion source type was H-ESI with a static spray voltage.

Unless otherwise stated, all solvents and reagents used for the extraction were high purity laboratory grade solvents from Carlo Erba (Milan, Italy). HPLC grade solvents water and acetonitrile were obtained from VWR (Milan, Italy) and used without further purification. Pure high purity analytical standards gallic acid, quercetin 3-*O*-glucoside, myricetin 3-*O*-rhamnoside and ginkolic acid 17:1 were purchased from Sigma (VWR Italia, Milan, Italy). Quantification of PLE metabolites was carried out via HPLC/DAD using the chromophore similarity principle: gallic acid was used to quantify itself and anacardic acid whilst all quercetin derivatives were quantified against quercetin 3-*O*-glucoside. Following the same method, all myricetin derivatives were quantified using myricetin 3-*O*-rhamnoside as external standard.

#### Prebiotic-like activity of PLE

The prebiotic-like property of PLE was tested on the probiotic strain *L. paracasei*. To assess the growth curves with PLE versus MRS medium standard, a bacterial suspension of 0.5 McFarland (1.5 × 10^8^ CFU/mL) was prepared, then a dilution was prepared to obtain a final concentration of 1.5 × 10^5^ CFU/mL in MRS broth. PLE was added in a concentration equal to 200 μg/mL (0.02% w/v). Inoculated 96-well polystyrene plates were incubated micro-aerobically with shaking at 37°C for 24 h, and the optical density measurements at 600 nm (BioTek Microplate Reader - Synergy HTX) were made every 30 min. Each determination was performed in triplicate within the same experiment, and the experiment was repeated three times. Moreover, inulin at a concentration (0.02% w/v) was included as a positive control for prebiotic activity. MRS broth containing only the bacterial inoculum was used as a growth control, whereas non-inoculated MRS broth without any additives served as a negative control to confirm medium sterility.

#### Growth conditions for production of Lp-CFS

The production of Lp-CFS from the tested strain was carried out following a modified version of the protocol proposed by Salemi *et al.*^70^ A total of 200 μL of bacterial suspension of 0.5 McFarland (approximately 1.5 x 10^8^ CFU/mL), were inoculated into 500 mL of MRS broth (resulting in a final *inoculum* of 0.04% (v/v) and incubated at 37°C for 48 h under microaerophilic conditions using GasPak (BD GasPakTM 100 Systems), to obtain a substantial bacterial pellet from the culture medium. After 48 h, the bacterial cultures were centrifuged at 6900 g (Centrifuge eppendorf 5810 R, Rotor F-34-6-38) to remove the medium. The resulting bacterial pellet was washed three times with sterile Dulbecco’s Phosphate Buffer Saline 1X (DPBS) to eliminate any residual medium. Subsequently, it was centrifuged again and resuspended in 500 mL of EMEM (ATCC-30-2003) supplemented with 1% L-Glutamine. The conditioning process lasted 24 h at 37°C under microaerophilic conditions. At this stage, the medium conditioned by bacterial growth was centrifuged 6900 g for 25 min at 4°C and then separated from the bacterial pellet by filtration through 0.45 μm sterile filters (Merck Millipore). After filtration, the pH of the medium was assessed to ensure its compatibility with cell culture experiments (pH 6.5–7.5).

#### Antibacterial activity

In agreement with CLSI M100^71^ agar well diffusion test was performed to evaluate antibacterial activity against pathogens. In this regard, 100 μL of the PLE solution at a concentration equal to 200 μg/mL was inoculated into wells in pre-inoculated MH agar plates with bacteria. For the Lp-CFS assay, 100 μL of conditioned EMEM medium was inoculated onto MH agar plates pre-seeded with bacteria. Similarly, for the Lp-CFS + PLE condition (200 μg/mL), 100 μL of the prepared mixture was added to the plates. All MH plates were incubated at 37°C overnight in aerobic conditions to evaluate antibacterial activity. Inhibition zones were measured by a caliper. Each determination was performed in triplicate within the same experiment, and the experiment was repeated three times. Results were expressed as mean ± SD.

#### DPPH assay

In order to evaluate the potential antioxidant activity of PLE, the DPPH assay was performed. This conventional test is based on the spectrophotometric measurement of the capacity of antioxidants to scavenge DPPH radicals.^72^ DPPH test spectrophotometrically measures the quenching ability of extracts at λ = 517 nm, as previously reported by Fuochi et al.^73^ Particularly, increasing concentrations of PLE included between (1000 - 10 μg/mL) were evaluated and compared to the standard control (Rutin 150 μg/mL). Each determination was performed in triplicate within the same experiment, and the experiment was repeated four times.

#### Cell line and organoids treatments

CCD18Co cell line was seeded in a 96-well polystyrene plate (Corning Costar Flat bottom cell culture microplates) to obtain a cell concentration of 1.0x10^5^ cells/well. After incubation, when 70% confluence was reached, the growth medium was eliminated to perform the assay to evaluate the cytotoxic activity of Lp-CFS (100-50%) or PLE 200 μg/mL. Cells incubated with culture medium only served as an untreated control. Mycoplasma test was performed using the MycoStrip kit (InvivoGen, rep-mys-50) and result was negative.

PDOs were seeded included in matrigel domes in 48-well plates (Corning Costar Flat bottom cell culture microplates) at a concentration of 40–80 organoids per dome. After 48 h, organoids were counted on optical microscope to perform the final data normalization.

Then, intestinal cultures (both 2D and 3D) were incubated for 48 h. PLE treatment was performed dissolving lyophilized PLE in growth medium to a final concentration of 200 μg/mL.

Moreover, in the last set of experiments, the LPS from *E. coli* (L2880, Sigma-Aldrich) at a concentration of 100 μg/mL was used as standard flogosis inducer while DMSO 5% v/v was selected as cytotoxic control producing cell damage.^74^ Every treatment was carried out for 24 h.

#### Evaluation of cytotoxicity of 2D and 3D cultures

Cytotoxicity evaluation was performed using the MTT assay.^75^ For both cell line and organoids culture, the working solution of MTT was prepared at a concentration of 5 mg/mL dissolving tetrazolium salts in DPBS. A proper volume of salts solution was added to each well to reach a final concentration of 0.5 mg/mL (1:10 dilution). Cultures were incubated for 30min-1 h and formazan crystals formation was monitored every 20 min. In the plate with cells, the supernatant was gently removed, formazan crystals were dissolved with 200 μL of DMSO per well. Instead, PDOs culture was subjected to an additional step after supernatant removal: 50 μL of Sodium Dodecyl Sulfate (SDS) 2% w/v was added and left to incubate for 20–30 min, so that domes dissolved. Then, after gently pipetting to dissolve eventual solid residues, 200 μL of DMSO was added to each well and the plate was incubated for 10–15 min. Finally, the optical density was measured in both cultures by spectrophotometer (BioTek Microplate Reader - Synergy HTX) at a wavelength of 562 nm.

#### Setup of the co-culture of PDOs and *L. paracasei*

The co-culture of PDOs and Lp was performed to validate the efficacy of PLE in a plausible biochemical context as close as possible to the intestinal niche. In this regard, PDOs were seeded at a concentration of 40–80 organoids (counting carried out with an optical microscope) per dome and incubated 48 h in growth medium. Then, a fresh solution of Lp equal to 0.5 McFarland was prepared and a volume of 20 μL was gently pipetted to each well to reach a final concentration of 1.5 × 10^7^ CFU/mL. The co-culture was monitored microscopically every 24 h and the percentage viability was quantified from 24 h up to one week. Each determination was performed in triplicate within the same experiment, and the experiment was repeated three times.

#### mRNA extraction and RT-qPCR

mRNA was extracted from each sample dissolving pellets in TRIzol (Invitrogen) to perform phenol-chlorophorm extraction method. The extracted mRNA was then converted to cDNA using the High-Capacity cDNA Reverse Transcription Kit (Thermo Fisher Scientific, Waltham, MA, USA). Quantitative Real-Time PCR was performed in Rotor-Gene Q2 (Qiagen) using PowerUp SYBR Green Master Mix (Applied Biosystems). The sequences of selected primers were listed in the **Primers Table**. For each sample, gene expression levels were normalized using β-actin as housekeeping gene. The relative mRNA expression was calculated using the comparative 2^−ΔΔCt^ method.^76^

**Table:** 

Primers Table			
Name	Sequence	Amp size (bp)	Accession numbers
IL10	Fw: CCACAAGACAGACTTGCAAAAG Rv: AACAAGTTGTCCAGCTGATCC	221	NM_000572.3
SOD1	Fw: AAAGATGGTGTGGCCGATGT Rv: CAAGCCAAACGACTTCCAGC	167	NM_000454.5
SOD2	Fw: CTGGACAAACCTCAGCCCTAAC Rv: AACCTGAGCCTTGGACACCAAC	137	NM_000636.4
TNFα	Fw: GCAACAAGACCACCACTTCG Rv: GATCAAAGCTGTAGGCCCCA	124	NM_000594.4
βactin Housekeeping gene	Fw: GCCTCGCCTTTGCCGAT Rv: AGGTAGTCAGTCAGGTCCCG	158	NM_001101.5

Description of the primer sequences expected amplicon sizes (bp), and GenBank accession numbers. Amplicon sizes and specificity were verified using NCBI Primer-BLAST. The housekeeping gene used is indicated. Primers were designed and validated using NCBI’s Primer-BLAST tool, with reference sequences identified by their respective accession numbers. Specificity was confirmed using the option in Primer-BLAST against the RefSeq mRNA database (organism: Homo sapiens). Primers were considered specific when no additional hits or unintended alignments were detected beyond the target gene.

### Quantification and statistical analysis

Data were analyzed using GraphPad Prism 10 (Version 10.4.0, Graph Pad Software, La Jolla, CA). Significant differences were designated as *p* < 0.05 and data were reported as mean ± SD for continuous variable, and as percentage for categorical variables. Prebiotic-like effect and cell viability of CCD18-Co treated with Lp-CFS_PLE were analyzed by two-way Analysis of Variance (ANOVA) followed by Tukey’s multiple comparison test. Moreover, DPPH, anti-inflammatory activity, and cytoprotective effect of PLE were analyzed by one-way ANOVA followed by Tukey’s multiple comparison test. Finally, cell viability of PDOs and CCD-18Co treated with PLE were analyzed using unpaired Student’s t test with two-tailed calculations of *p*-value. All data passed normality testing. Statistical analyses for outliers (Grubbs’ test) were conducted on all datasets, and no outliers were identified. *p* < 0.05 was considered significant, marked as ∗; *p* < 0.01: ∗∗, *p* < 0.001: ∗∗∗, *p* < 0.0001: ∗∗∗∗.

For quantification of PLE metabolites, limit of detection LOD and limit of quantification LOQ values were reported for the analytical standards in Quantification of PLE metabolites Table. Calculated LOD e LOQ were expressed in mg/mL, considering S/*N* 3.3 and 10 respectively.

**Table:** 

Quantification of PLE metabolites table			
	Quercetin 3-*O*-glucoside	Gallic acid	Myricetin 3-*O*- rhamnoside
σ	4.529994481	17.39170057	14.39265184
CRL	15418516.09	3731360.304	15373692.26
LOD	1.4096 × 10^−5^	3.8358 × 10^−6^	3.1881 × 10^−7^
LOQ	4.2716 × 10^−5^	1.1623 × 10^−5^	9.6611 × 10^−7^
R2	0.9999	0.9995	0.9989
